# Who is leading medical AI? A systematic review and scientometric analysis of chest x-ray research

**DOI:** 10.64898/2026.04.02.26349884

**Published:** 2026-04-07

**Authors:** Constanza Vásquez-Venegas, Api Chewcharat, Richard Kimera, Nicholas Kurtzman, Marianna Leite, Naira Link Woite, Ishvica J. Muppidi, Rachel J. Muppidi, Xioli Liu, Erika P. Ong, Ridhima Pal, Christopher Myers, Sebastian Salzman, Jonathan Sønderbo Patscheider, Taniya Raji John, Mwavu Rogers, Mathew Samuel, Juan Luis Santana-Guerrero, Shahar Yaacob, Rodrigo Gameiro, Leo A. Celi

## Abstract

Computer vision models for chest X-ray interpretation hold significant promise for global healthcare, but their clinical value depends on equitable development across diverse populations. We conducted a scientometric analysis to examine authorship patterns, geographic distribution, and dataset origins to assess potential disparities that could affect clinical applicability.

We systematically reviewed literature on computer vision applications for chest X-rays published between 2017-2025 across multiple databases, including PubMed, Embase and SciELO databases. Using Dimensions API and manual extraction, we analyzed 928 eligible studies, examining first and senior author affiliations, institutional contributions, dataset provenance, and collaboration patterns across different income classifications based on World Bank categories.

High-income countries dominated research leadership, representing 55.6% of first authors and 59.7% of senior authors; no first authors were affiliated with low-income countries.

China (16.93%) and the United States (16.72%) led in first authorship positions. Most datasets (73.6%) originated from high-income settings, with the United States being the largest contributor (40.45%). Private datasets were most frequently used (20.52%). Cross-income collaborations were rare, with only 3.9% of publications involving partnerships between high-income and lower-middle-income countries.

Findings reveal substantial disparities in who shapes computer vision research on chest X-rays and which populations are represented in training data. These imbalances risk developing AI systems that perform inconsistently across diverse healthcare settings, potentially exacerbating healthcare inequities. Addressing these disparities requires coordinated efforts to develop globally representative datasets, establish equitable international collaborations, and implement policies that promote inclusive research practices.

**Author Summary:**

In this study, we examined the global landscape of research involving computer vision applied to chest X-rays. While these technologies have the potential to significantly improve healthcare worldwide, their effectiveness depends on being developed and tested using data from diverse populations. We analyzed nearly one thousand scientific articles and found that research leadership and data sources are heavily concentrated in high-income countries, particularly the United States and China.

Our findings reveal a concerning gap because people in low-income regions, who often face the highest burden of respiratory diseases, are almost entirely absent from the research process as both authors and data contributors. This imbalance creates a risk that medical artificial intelligence may not perform reliably when used in different parts of the world, which could accidentally worsen existing health inequalities. We argue that the scientific community must prioritize international partnerships that treat researchers from developing nations as equal leaders. By making medical data more diverse and accessible, we can ensure that these powerful diagnostic tools benefit patients everywhere, regardless of their location or economic status.

## Full Text Availability

The license terms selected by the author(s) for this preprint version do not permit archiving in PMC. The full text is available from the preprint server.

